# Assessing Semiregional Cerebral Oxygen Consumption (CMRO_2_) in Preterm Neonates: A Quantitative MRI Cohort Study With Exploratory Analysis of Respiratory Support

**DOI:** 10.1002/nbm.70065

**Published:** 2025-05-14

**Authors:** Chen Shuang Zhu, Natalie Chan, Anil Chacko, Liisa Holsti, Ruth E. Grunau, Alexander Mark Weber

**Affiliations:** ^1^ School of Biomedical Engineering The University of British Columbia Vancouver British Columbia Canada; ^2^ BC Children's Hospital Research Institute The University of British Columbia Vancouver British Columbia Canada; ^3^ Department of Pediatrics University of California San Francisco San Francisco California USA; ^4^ BC Women's Hospital Vancouver British Columbia Canada; ^5^ Occupational Science & Occupational Therapy University of British Columbia Vancouver British Columbia Canada; ^6^ Department of Pediatrics The University of British Columbia Vancouver British Columbia Canada

**Keywords:** arterial spin labeling, cerebral blood flow, cerebral metabolic rate of oxygen, preterm, quantitative susceptibility mapping, respiratory support, ventilation

## Abstract

Developing a noninvasive method for measuring oxygen consumption at both regional and whole‐brain levels in preterm infants is crucial for assessing brain development and neuronal injury in this vulnerable population. This study presents a multi‐modal MRI technique and analysis pipeline that produces whole‐brain semiregional maps—with the potential to be fully regional—which we employ in a cohort study to investigate how the duration of various respiratory supports in very preterm infants affects CBF and the cerebral metabolic rate of oxygen (CMRO_2_). Infants (*n* = 19) born < 32 weeks gestational age were recruited in the neonatal intensive care unit. Infants were scanned at term‐equivalent age using a 3 T MRI sequence comprising T_1_‐weighted, T_2_‐weighted, arterial spin labeling (ASL), and SWI. Days on three different categories of respiratory support, based on levels of invasiveness, were recorded. Using multiple linear regression, CBF and CMRO_2_ were analyzed against days on: respiratory support, days in room air, and the proportion of days on respiratory support; GA and PMA were used as confounding factors. Average CBF and CMRO_2_ of cortical grey matter was 14.3 ± 4.25 mL/100 g/min and 29.49 ± 29.49 μmol/100 g/min, respectively. CMRO_2_ and CBF were positively correlated with days on noninvasive respiratory support and negatively correlated with days in room air. Using our novel method, CBF and CMRO_2_ values aligned closely with literature values. Our exploratory findings suggest that the type of respiratory support may influence cerebral oxygenation during the neonatal period in infants born very preterm, with greater oxygen delivery and consumption associated with noninvasive respiratory support. Our semiregional brain analysis further highlights that different brain structures are impacted in distinct ways. This study presents a novel multimodal MRI approach to measure cerebral blood flow (CBF) and oxygen metabolism (CMRO_2_) in preterm infants. Exploratory findings suggest noninvasive respiratory support is associated with higher CBF and CMRO_2_, highlighting its potential impact on neonatal brain oxygenation and development.

## Graphical abstract

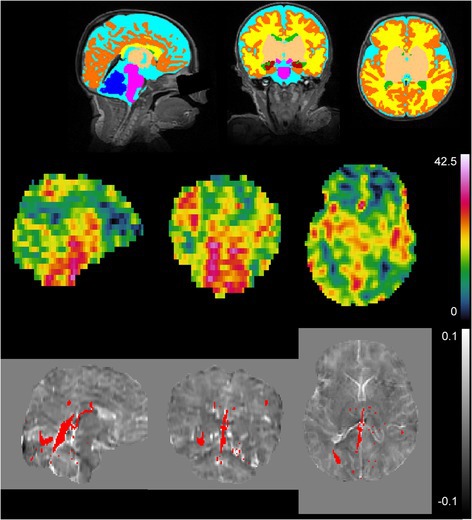

**Abbreviations**
ASL: arterial spin labeling
CMRO_2_
: cerebral metabolic rate of oxygen
CSaO_2_
: cerebral arterial oxygen saturation
CSvO_2_
: cerebral venous oxygen saturation
GA: gestational age
CGM: cortical grey matter
DGM: deep grey matter
Hct: hematocrit
NICU: neonatal intensive care unit
NIRS: near‐infrared spectroscopy
OEF: oxygen extraction fraction
PMA: post‐menstrual age
PPM: parts per million
QSM: quantitative susceptibility mapping
T_2_‐TRIR: T_2_ Prepared Tissue Relaxation Inversion Recovery
TRUST: T_2_‐relaxation‐under‐spin‐tagging

## 1 Introduction

The developing brains of preterm infants are vulnerable to injury and dysmaturation, which can result in long‐term neurological deficits [1]. Infants born very preterm (≤ 32 weeks gestation) are particularly at risk for significant short and long‐term respiratory problems, and the lower the gestational age at birth, the more likely the infant may be negatively impacted [2]. To mitigate these risks, it is crucial to monitor brain hemodynamics, as instability in oxygen delivery and metabolism can contribute to these injuries [3]. These infants are also at significant risk of respiratory disorders, such as respiratory distress syndrome [4], and bronchopulmonary dysplasia [5]. Finding ways to prevent these respiratory disorders, and support lung development, is critically important as lung health helps determine the amount of oxygen the brain receives [6, 7]. Therefore, close monitoring of cerebral oxygenation is critical to identify these risks early and apply neuroprotective strategies that may mitigate long‐term neurological consequences.

In the NICU, to aid in respiration and improve lung function, various strategies including different forms of ventilation can be used, where the general goal is to decrease days of invasive mechanical ventilation. Noninvasive modes of ventilation, such as continuous positive airway pressure and nasal intermittent positive pressure ventilation, have been shown to be effective in lowering rates of complications and mortality compared with intubation with mechanical ventilation [8, 9, 10, 11]. With advances in neonatal intensive care and the use of less invasive forms of respiratory support, the incidence of bronchopulmonary dysplasia and other respiratory complications in preterm neonates has decreased over time [12]. However, the optimal mode and timing of ventilation in preterm neonates with respiratory disorders are still being debated [13, 14, 15, 16].

Noninvasive MRI‐based techniques are actively being explored to assess whole‐brain oxygen consumption. One approach combines noninvasive venous oxygenation measurements from the sagittal sinus using T_2_‐relaxation‐under‐spin‐tagging magnetic resonance imaging (TRUST [17]) with flow measurements from phase‐contrast MR angiography, which has been used in adults [18] and has shown feasibility in neonates [19, 20]. Another method combines MR susceptometry to measure venous oxygenation in the sagittal sinus with phase‐contrast MR angiography [21]. Studies using these techniques in neonates demonstrated that the results correlate well with those obtained through diffuse optical and correlation spectroscopy methods [22]. Still another method applied in neonates is the T_2_ prepared tissue relaxation inversion recovery (T_2_‐TRIR) MRI pulse sequence [23], which measures the transverse and longitudinal relaxation rate of blood (T_2b_ and T_1b_) in the sagittal sinus, and venous oxygenation subsequently derived from the T_2b_ and the T_1b_‐derived hematocrit [24].

In the current study, we propose a new method using quantitative susceptibility mapping (QSM) and arterial spin labeling (ASL)—in combination with hematocrit (Hct) and pulse oximetry—to determine whole‐brain semiregional cerebral metabolic rate of oxygen (CMRO_2_) and regional CBF. In order to investigate the validity of this new noninvasive approach we compared the obtained results to previously reported reference values [19, 20, 25, 26, 27, 28, 29]. In addition, we conducted an exploratory analysis to evaluate if the technique can detect whether the degree of lung disease, as indicated by the duration of time on different levels of respiratory support in very‐preterm neonates, is correlated with brain oxygenation measures CMRO_2_ and CBF in different brain regions at term‐equivalent age. We hypothesized the CMRO_2_ and CBF would be negatively correlated with time on invasive respiratory support.

## 2 Methods

### 2.1 Strobe

The methodology and its reporting have followed the STrengthening the Reporting of OBservational studies in Epidemiology (STROBE) standards. We include the checklist for a cohort study in our Supplementary Materials.

### 2.2 Patients

The study was approved by the Clinical Research Ethics Board at the University of British Columbia and Children's & Women's Hospital (H21‐00655) and written informed consent was obtained from the parent/guardian for each infant.

Preterm neonates born < 32 weeks gestational age (GA) (*n* = 20) admitted to the level III neonatal intensive care unit (NICU) at BC Women's Hospital (BCWH) in Vancouver Canada were recruited by a research nurse from February 2021 to January 2022. Our sample size was determined based on similar recruitment numbers from previous studies [19, 20, 25, 26, 27, 28, 29]. Inclusion criteria were infants born less than 32 weeks GA. Infants were excluded if there was evidence of a congenital malformation or syndrome, a TORCH infection, or ultrasound evidence of large periventricular hemorrhagic infarction (> 2 cm, Grade 4 intraventricular hemorrhage). Parents were approached by the research nurse about the study shortly before being discharged from the NICU. Infants returned to the hospital for the study at TEA (37–44 weeks PMA) for the MRI scan. One infant remained in hospital on respiratory support at 44 weeks PMA, and was withdrawn from the study. The final number of infants scanned was 19. The clinical characteristics of the subjects are shown in Table 1.

**TABLE 1:** Neonatal and maternal characteristic of the study sample.

Maternal characteristics (*n* = 19)		Neonatal characteristics (*n* = 19)	
Gestational diabetes	6 (31.6%)	Male (*n*)	10 (52.6%)
***Delivery mode***		Female (*n*)	9 (47.4%)
Cesarean	16 (84.2%)	Birth weight (g)	1304 (1054.5–1484.5)
Vaginal	3 (15.8%)	GA at birth (weeks)	28.86 (27.79–29.93)
***Maternal fever***		PMA on scan day (weeks)	40.57 (39.29–41.36)
Yes	1 (5.3%)	Weight on scan day (g)	3330 (2990–3910)
No	6 (31.6%)	Head circumference on scan day (cm)	35.50 (33.75–36.00)
Unknown	12 (63.2%)	Days in NICU	53 (37–60)
***Chorioamnionitis***		Days on sedatives	0 (0–0)
Yes	4 (21%)	Days on narcotic infusion	1 (0–2)
No	12 (63.2%)	Days on Category 1 (invasive ventilation)	2 (0–4)
Unknown	3 (15.8%)	Days on Category 2 (noninvasive ventilation)	19 (11.5–32)
***Leukocytosis***		Days on Category 3 (high‐flow/low‐flow)	7 (5.5–12)
Yes	1 (5.3%)	Total days on respiratory support	31 (23–51.5)
No	4 (21%)	Days in room air	11 (3.5–23)
Unknown	14 (73.7%)		
Gestational hypertension	3 (15.8%)		
Pre‐existing hypertension	1 (5.3%)		
***Systemic antibiotics***			
Yes	15 (78.9%)		
No	1 (5.3%)		
Unknown	3 (15.8%)		

*Note:* Median (Q1–Q3) is shown for continuous variables and *n* (%) for categorical variables.

Abbreviations: GA = gestational age at birth; PMA = postmenstrual age on the day of the scan.

### 2.3 MRI Data Acquisition

All scans took place at the BC Children's MRI Research Facility and were performed on a 3.0 Tesla General Electric Discovery MR750 (scanner software version DV26.0_R03) with a SREE Medical Systems single channel neonatal head coil. All scans were research‐dedicated and not research sequences added on to clinical scans. Infants were fed and swaddled by a research nurse prior to being placed in an MRI compatible incubator for imaging (SREE Medical Systems). Molded foam was placed around the infant's body and head to minimize head movement and ear plugs were used for ear protection. Arterial oxygen saturation (CSaO_2_) and heart rate were monitored and measured continuously throughout the scan using a pulse oximeter placed on the foot of the infant. A neonatologist (NC or AC) and primary investigator (AMW) were present throughout the scan.

MRI scans were performed using a protocol consisting of a T_1_‐weighted scan, a T_2_‐weighted scan, a pseudo‐continuous ASL scan to measure CBF, an SWI scan to generate QSM maps, and a diffusion weighted imaging spin echo EPI sequence (not included in this report). Sequences were repeated when large motion artifacts were detected. If an infant awoke or was moving during the scan, the scanning was stopped, and a research nurse entered the MRI room to monitor and ensure the infant fell back to sleep.

The T_1_‐weighted coronal 3D‐FSPGR parameters were: 2.97 ms TE, 7.74 ms TR, 12° flip angle, 20 cm FOV, 512 × 512 matrix, 0.39 × 0.39 mm in‐plane resolution, 1 mm slice thickness, 126 slices, and a scan duration of 4 min 39 s. The T_2_‐weighted sagittal 3D‐CUBE parameters were: 66.29 ms TE, 2300 ms TR, 90° flip angle, 20 cm FOV, 256 × 256 matrix, 0.78 × 0.78 mm in‐plane resolution, 1 mm slice thickness, 106 slices, and a scan duration of 5 min 1 s.

The pseudo‐continuous ASL axial multishot spiral 3D fast spin‐echo parameters were: 10.55 ms TE, 4.68 s TR, 111° flip angle, 24 cm FOV, 128 × 128 matrix, 1.875 × 1.875 mm in‐plane resolution, 4 mm slice thickness, 25 slices, 1450 ms label period, 2025 ms post‐labeling delay, 24 control‐label pairs, and a scan duration of 5 min 26 s.

The SWI axial 3D spoiled GRE flow compensated parameters were: five equally spaced echoes, 5 ms first TE, 5.24 ms echo spacing, 30.9 ms TR, 20° flip angle, 25 cm FOV, 256 × 256 matrix, 0.977 mm in‐plane resolution, 2 mm slice thickness reconstructed to 1 mm using zero‐padded Fourier interpolation (ZIP2), 46 slices, and a scan duration of 5 min 29 s.

A DWI spin‐echo EPI sequence was also acquired, but was not analyzed for this study.

### 2.4 Clinical Data Collection

Hct values were acquired retrospectively from chart review. Hct values for the day of scan were predicted using a four‐parameter Weibull function (drc; fct = W1.4; Figure S1). Clinical variables were obtained from the Canadian Neonatal Network (CNN). Days on respiratory support were categorized into three groups: Category 1 (invasive ventilation) included high frequency jet ventilation, high frequency oscillatory ventilation, and intermittent positive‐pressure ventilation (either volume or pressure targeted); Category 2 (noninvasive ventilation) included noninvasive positive pressure ventilation and continuous positive airway pressure; and Category 3 included high‐flow and low‐flow nasal cannula. The three categories represented the invasiveness of respiratory support, with Category 1 being the highest, and Category 3 being the lowest.

### 2.5 MRI Data Processing

Imaging data was processed using an in‐house pipeline written in Bash shell script by A.C. with minor edits by A.M.W. (https://github.com/WeberLab/CMRO2). Large portions of the pipeline use the FMRIB Software Library (FSL; v6.0) [30]. A csv file consisting of all the subject IDs, age in weeks, and the folder numbers of the raw imaging data was used as an input to the pipeline. A dcm2bids [31] configuration JSON file was created using information from the input file which assigns the raw imaging DICOM files a data type, modality label, phase encoding direction for spin echo field maps, sidecar file name which corresponds to the chronological order of the scan acquisition, and sidecar changes. The dcm2bids tool used Chris Rorden's dcmniix [32] to convert the raw DICOM files to NIfTI (Neuroimaging Informatics Technology Initiative) format and organized the data according to the Brain Imaging Data Structure (BIDS) standard format [33].

The T_1_w and T_2_w files were then processed using the dHCP structural pipeline [34]. The dHCP structural pipeline is a specialized neonatal pipeline that automates registration, segmentation, surface extraction, and surface registration of structural MRI images of the neonatal brain. A docker container was set up to run the dHCP structural pipeline using the subject ID, age in weeks, T_1_w, and T_2_w images. The dHCP pipeline first uses FSL's bet [35] to perform an initial skull‐stripping with a fractional intensity threshold of 0.1. Tissue segmentation is then accomplished using the DRAW‐EM algorithm [36], which uses an atlas‐based segmentation technique and an expectation–maximization scheme that combines the structure priors and an intensity model of the volume.

Tissue labels were then thresholded and binarized using FSL's fslmaths to create a total of nine masks. These regions included the CSF, cortical grey matter (CGM), WM, background, ventricles, cerebellum, deep GM (DGM), brainstem, and the hippocampus and amygdala as one mask.

QSM imaging data of all five echoes was then post‐processed from the phase data using a script from Christian Kames (github.com/kamesy/QSM.m) [37] which follows the recommended implementation of QSM for clinical research in the brain [38]. A brain mask from the fifth echo magnitude image was obtained using FSL's bet. Improved brain masks were obtained from the square of the magnitude image (fslmaths‐sqr). A two‐voxel kernel was used to erode the brain mask. Phase unwrapping was achieved using a 3D Laplacian algorithm and background field removal was completed by fitting a fourth order 3D polynomial to the unfiltered phase data. A Gaussian filter (σ = 0.5) was applied to the normalized field maps to smooth out high frequency errors from reconstruction. A two‐step dipole inversion algorithm was used to solve the dipole inversion problem [39]. Fslroi and fslmaths were then used to retrieve the average of the last three echoes. R_2_* maps were calculated by performing a linear fit of the logarithm of the magnitude values against the TEs. Finally, vascular masks were created using Sina Straub's GRE_vessel_seg (github.com/SinaStraub/GRE_vessel_seg) [40] with elements borrowed from Warda‐Taqdees Syeda's QSMART (textgithub.com/wtsyeda/QSMART) [41, 42, 43, 44]; both of which make use of Chunlei Liu's STI Suite [45]. Default parameter settings were used including a recursive ridge filter and a scale number (kernel size) equal to 4 [46]. The χ of cerebral veins was then measured by thresholding out values below 0.15 ppm (ppm) [47, 48] and averaged within the vascular mask to find the mean of nonzero voxels. CSvO_2_ can then be calculated using the averaged χ value and the following equation [49]:

(1) $$\mathrm{CSv}O_{2}=1-\frac{\left(\Delta \chi_{\text{blood}}-\Delta \chi_{\mathrm{oxy}}\cdot \mathrm{Hct}\right)}{\left(\Delta \chi_{\mathrm{do}}\cdot \mathrm{Hct}\right)}$$

where CSvO_2_ is the cerebral venous oxygen saturation, $\Delta \chi_{\text{blood}}$ is the measured susceptibility difference between blood and water, $\Delta \chi_{\mathrm{oxy}}$ is the susceptibility changes of oxygenated red blood cells relative to water, $\Delta \chi_{\mathrm{do}}$ is the susceptibility difference between oxygenated and deoxygenated red blood cells, and Hct is the hematocrit value. $\Delta \chi_{\text{blood}}$ was the averaged χ of cerebral veins minus the average χ of CSF; $\Delta \chi_{\mathrm{oxy}}$ was −0.21*4 ppm as per Sedlacik et al. [50] and Portnoy et al. [51]; $\Delta \chi_{\mathrm{do}}$ was −0.03*4 ppm as per Weisskoff and Kiihne [49]; and Hct was predicted based on previous measured values for the day of the scan.

Oxygen extraction fraction (OEF) was calculated as

(2) $$\mathrm{OEF}=\left(\mathrm{CSa}O_{2}-\mathrm{CSv}O_{2}\right)/\mathrm{CSa}O_{2}$$

where CSaO_2_ was calculated from the pulse oximeter during the MRI scan and CSvO_2_ was calculated from Equation (1).

ASL imaging data were then post‐processed in the pipeline using an in‐house python script to generate a CBF map https://github.com/WeberLab/CMRO2. The imaging data consisted of two images, a PWI which is the difference between the control and labeled images, and a proton density image which is used for scaling signal intensities. The python script uses the PWI and proton density images along with other neonatal specific parameters derived from the literature and the MRI acquisition parameters as inputs and generates a CBF map with the following quantification model:

(3) $$\mathrm{CBF}=6000\cdot \lambda \frac{\left(1-\exp\left(-\frac{\mathrm{ST}\left(s\right)}{T_{1t}\left(s\right)}\right)\right)\exp\left(\frac{\mathrm{PLD}\left(s\right)}{T_{1b}\left(s\right)}\right)}{2\cdot T_{1b}\left(s\right)\cdot \left(1-\exp\left(-\frac{\mathrm{LT}\left(s\right)}{T_{1b}\left(s\right)}\right)\right)\cdot \epsilon \cdot \mathrm{NE}X_{\mathrm{PW}}}\left(\frac{\mathrm{PW}}{SF_{\mathrm{PW}}\cdot \mathrm{PD}}\right)$$

where CBF is the cerebral blood flow in mL/100 g/min, λ is the blood/tissue water partition coefficient set to 0.9 mL/g as per Alsop et al. [52], T_1b_ is the longitudinal relaxation time of blood and was set to 1.89 s as per De Vis et al. [53], T_1t_ is the longitudinal relaxation time of tissue and was set to 1.2 s, ST is the saturation time (2 s), LT is the labeling duration (1.45 s), PLD is the labeling delay (2.025 s), ϵ is the efficiency set to 0.6, NEX_pw_ is the number of excitations for PWI images (3), SF_pw_ is the PWI sequence scaling factor (32), PW is the perfusion weighted image, and PD is the proton density image. The constant 6000 converts the unit from mL/g/s to mL/100 g/min.

The volume of the tissue masks for each ROI was calculated using fslstats. The tissue masks were registered to the averaged χ and CBF map by applying the mask and taking the mean of the whole image. All generated variables including the ROI volumes and averages in tissue masks were output into a comma separated variable file with the subject ID and headers for each corresponding column.

Using values from the csv file, the following equation was used to quantify CMRO_2_ in R:

(4) $$\mathrm{CMR}O_{2}=\mathrm{OE}\cdot \mathrm{CBF}\cdot \mathrm{HbT}=\left(\mathrm{CSa}O_{2}-\mathrm{CSv}O_{2}\right)\cdot \mathrm{CBF}\cdot \mathrm{HbT}$$

where CMRO_2_ is the cerebral metabolic rate of oxygen in μmol/100 g/min, OE is the oxygen extraction (the difference between CSaO_2_ and CSvO_2_), CBF is the cerebral blood flow in mL /100 g /min, HbT is the hemoglobin concentration and is generally assumed to be Hct/(3 mL/g*0.01625 g/μmol) [54, 55]. Semiregional CMRO_2_ and regional CBF values were calculated by averaging within specific ROIs.

### 2.6 Statistics

Statistical analysis was performed using R (v. 4.4.3) [56] and R studio (v. 2022.12.0 Build 353) [57]. A multiple linear regression analysis was conducted to examine relationships between dependent variables CBF or CMRO_2_ and independent variables (e.g., days on Category 1 support). GA at birth and post‐menstrual age (PMA) at time of scan were included as confounding factors. The correlation coefficient, *p*‐value, and beta value were determined for each individual analysis. Significant relationships were considered with a *p*‐value of 0.05. Multiple comparison corrections were not applied as our analysis was primarily exploratory.

Using a multiple linear regression analysis including GA and PMA as confounding factors, CBF and CMRO_2_ were analyzed separately against days on: the three separate categories of respiratory support, the number of days in room air (total days in the NICU minus total days on respiratory support), and the proportion of days on respiratory support (total days on respiratory support divided by total days in the NICU). Previous studies have shown a significant negative relationship between the brainstem volume of very preterm neonates at TEA and prolonged days on mechanical ventilation [7]; thus the days on Category 1 respiratory support was analyzed with brainstem volumes.

## 3 Results

**FIGURE 1:**
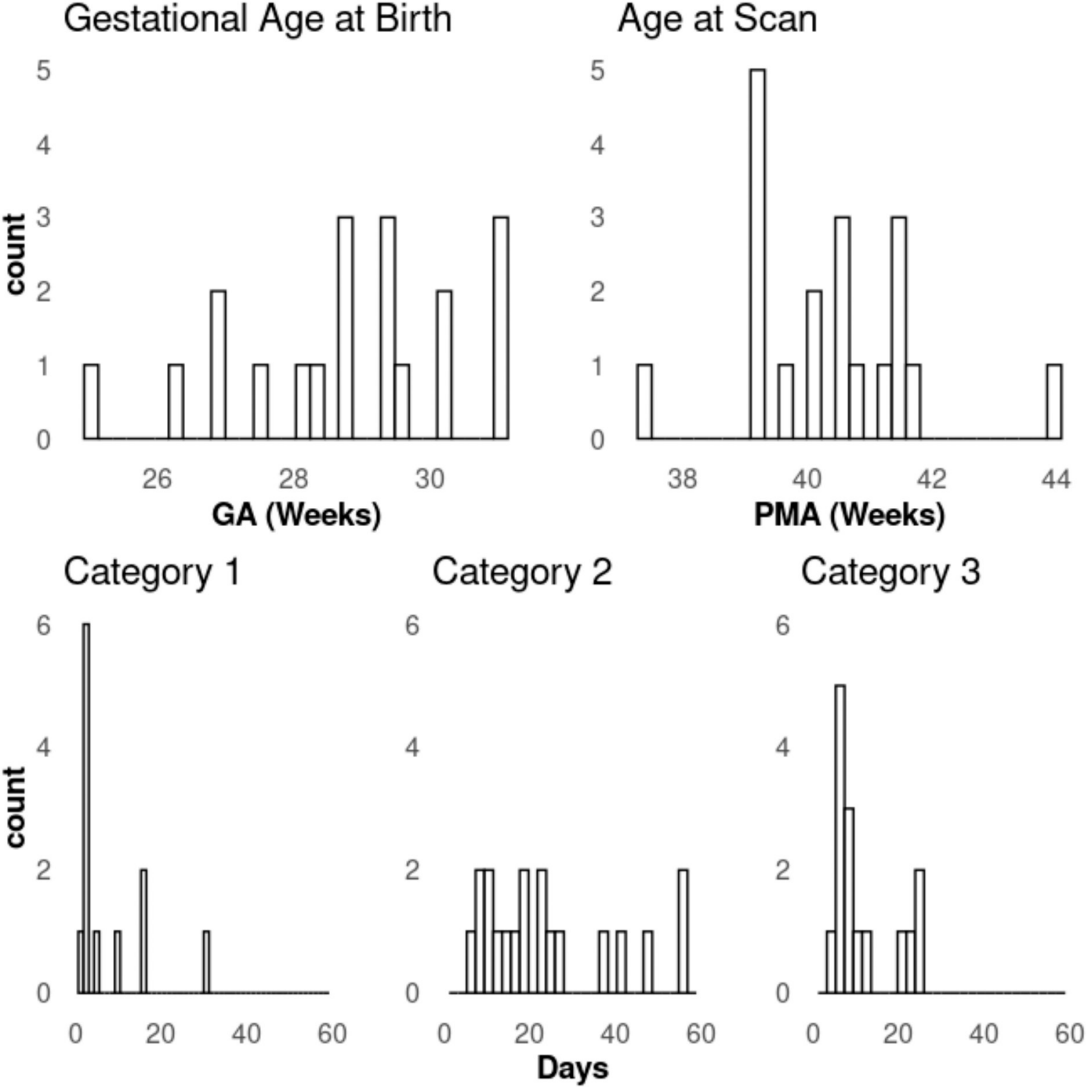
Types of respiratory support in relation to gestational age at birth, post‐menstrual age (PMA) at scan, and days on each type of respiratory support. *Note:* counts of 0 days on respiratory support are not shown. Category 1 = invasive ventilation; category 2 = noninvasive ventilation; category 3 = high‐flow and low‐flow support.

Median (Q1–Q3) GA at birth and PMA at scan time were 28.86 (27.79–29.93) and 40.57 (39.29–41.36) weeks, respectively. Median (Q1–Q3) days on Categories 1, 2, and 3 respiratory support, in room air, and length of stay in the NICU were 2 (0–4), 19 (11.5–32), 7 (5.5–12), 11 (3.5–23), and 53 (37–60), respectively (Table 1 and Figure 1).

**FIGURE 2:**
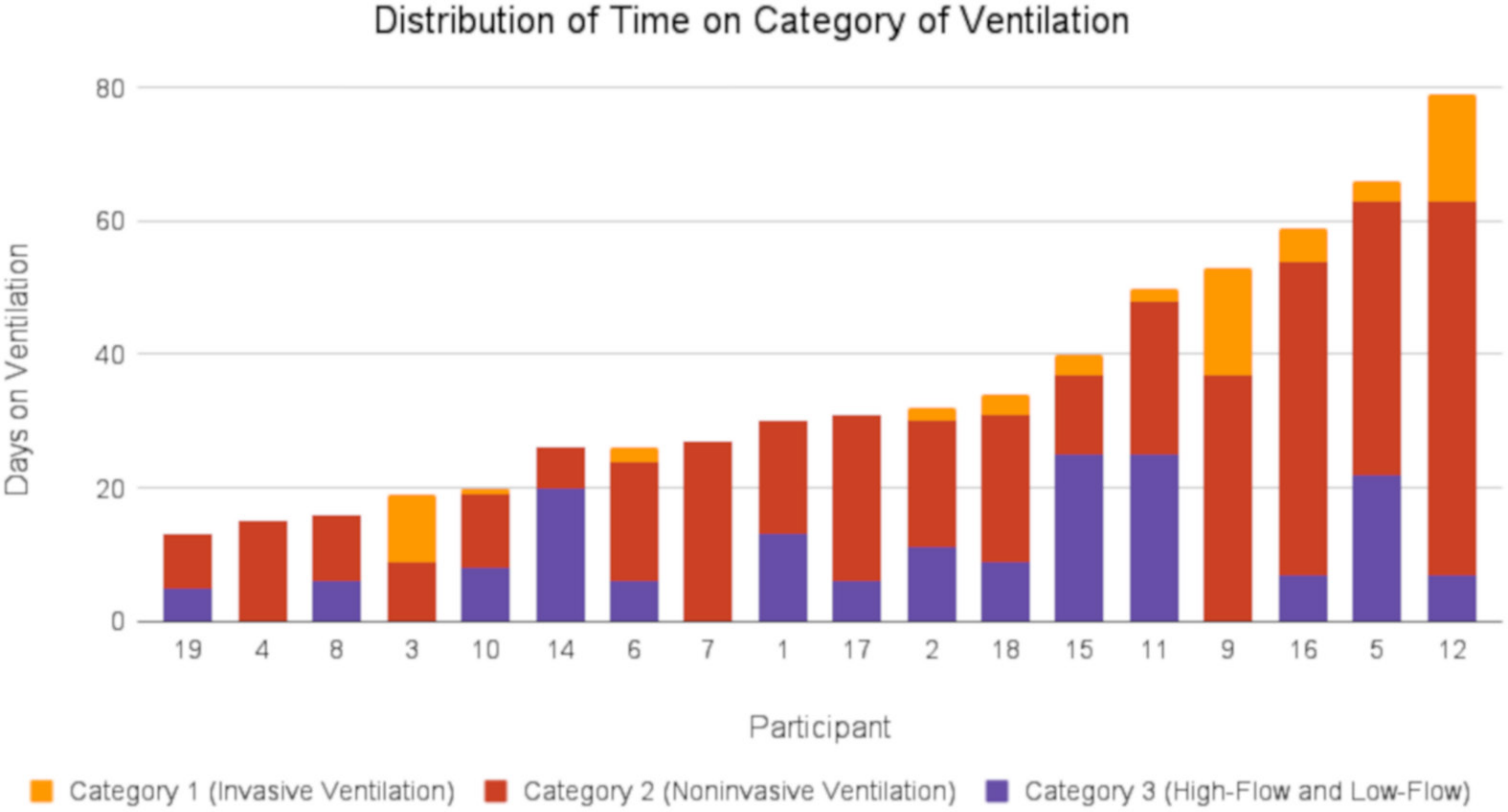
Days on the three levels of respiratory support for each study subject.

A subject‐by‐subject distribution of days on different categories of respiratory support is shown in Figure 2.

**FIGURE 3:**
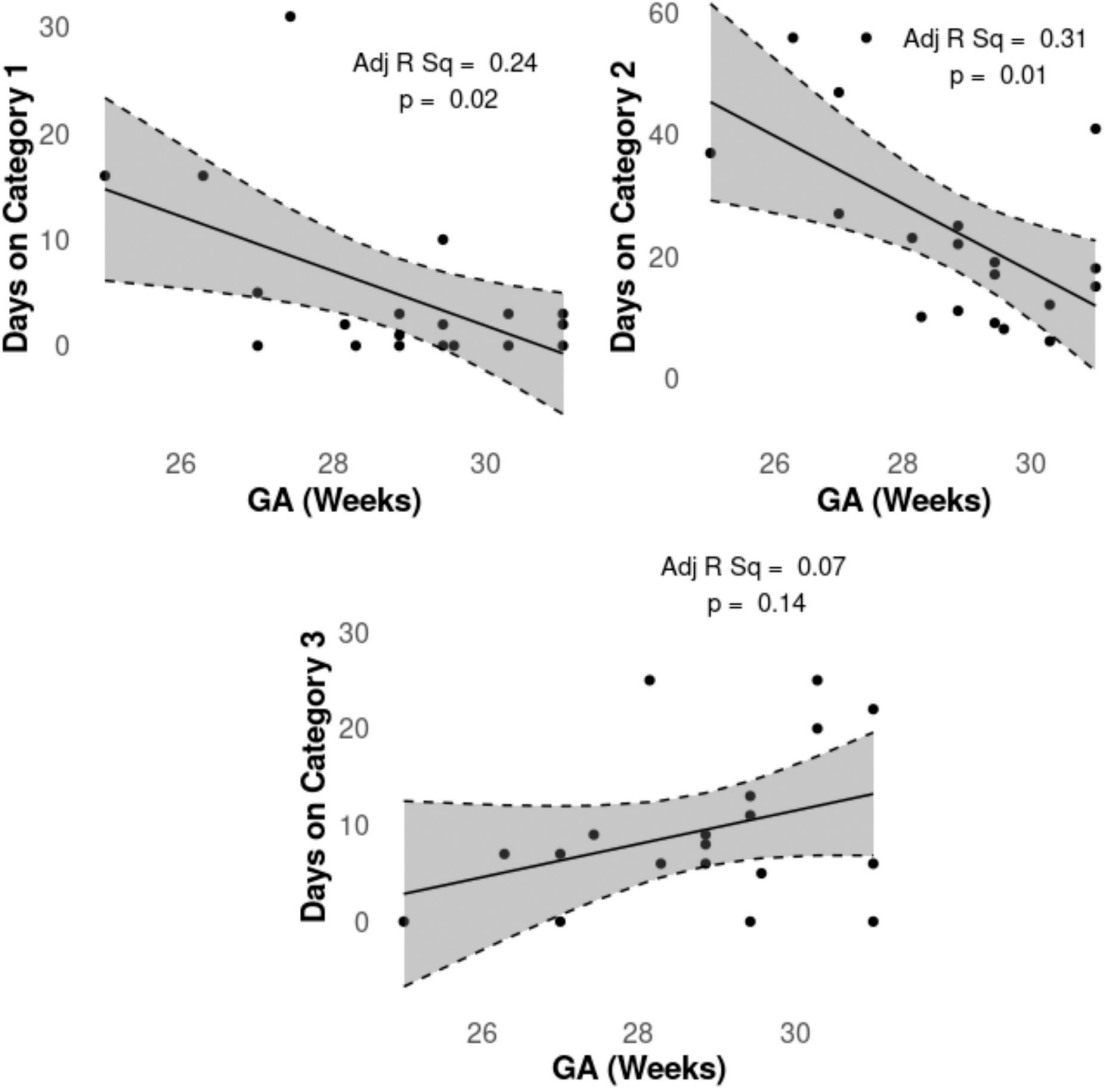
Linear regression of days on the three categories of respiratory support vs gestational age.

Gestational age at birth was found to be negatively correlated with both days on Category 1 (invasive ventilation) and Category 2 (noninvasive ventilation) respiratory support, but not Category 3 (high‐flow/low‐flow; Figure 3).

**FIGURE 4:**
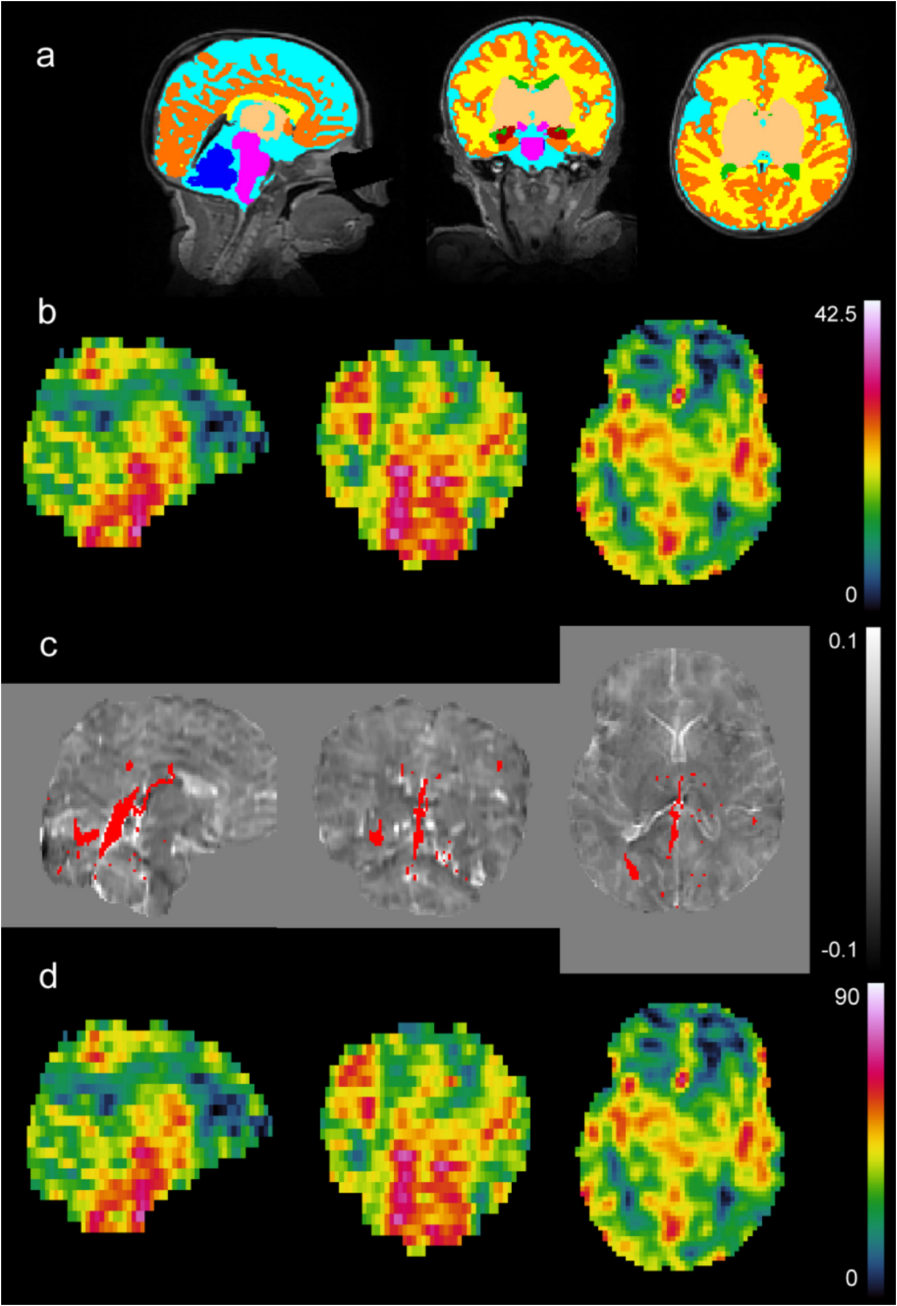
Sample images from a subject of various MRI results. All images show a sagittal, coronal and axial slice from left to right. Panel (A) is a T_2_w image in the background with segmentation overlaid in various colors: dark blue = cerebellum; pink = brainstem; light blue = CSF; yellow = white matter; dark orange = cortical grey matter; green = ventricles; light orange = deep grey matter; dark red = hippocampus and amygdala. Panel (B) is a processed CBF map from 0 to 47.5 mL/100 g/min. Panel (C) is a QSM image in the background from −0.1 to 0.1 ppm susceptibility overlaid with a venous mask in red. D) is a processed CMRO_2_ image from 0 to 93 μmol/100 g/min. Note that panels (B) and (D) look identical as panel (D) is simply panel (B) multiplied by a value determined by CSaO_2_, CSvO_2_, and Hct (see Equation 4). However, this value will be different for every subject.

A sample of results of the MRI analysis, including a sample brain segmentation, CBF map, QSM map, and CMRO_2_ map are shown in Figure 4.

Mean whole‐brain CSvO_2_, CSaO_2_, Hct and OEF values were 63.9 ± 4%, 98.3 ± 1.5%, 29.7 ± 3.5%, and 34.9 ± 4.3%, respectively. Semiregional mean CMRO_2_ and regional CBF and values are shown in Table 2. The lowest CBF and CMRO_2_ values were found in the WM, while the highest CBF and CMRO_2_ values were found in the brainstem.

**TABLE 2:** Semiregional CMRO_2_ and regional CBF mean ± standard deviation values.

ROI	CBF (mL/100 g/min)	CMRO₂ (μmol/100 g/min)
CGM	14.3 ± 4.25	29.49 ± 8.62
WM	11.18 ± 3.11	23.08 ± 6.47
DGM	18.1 ± 6.63	37.22 ± 13.23
Brainstem	27.16 ± 11.05	55.69 ± 21.34
Cerebellum	21.78 ± 8.27	44.68 ± 15.98
Hippocampus and amygdala	19.98 ± 6.85	41.14 ± 13.69

*Note:* White matter tissue was found to have the lowest CBF/CMRO_2_ values, while the brainstem had the highest.

Abbreviations: CBF = cerebral blood flow; CGM = cerebral grey matter; CMRO₂ = cerebral metabolic rate of oxygen; DGM = deep grey matter; ROI = region of interest; WM = white matter.

**FIGURE 5:**
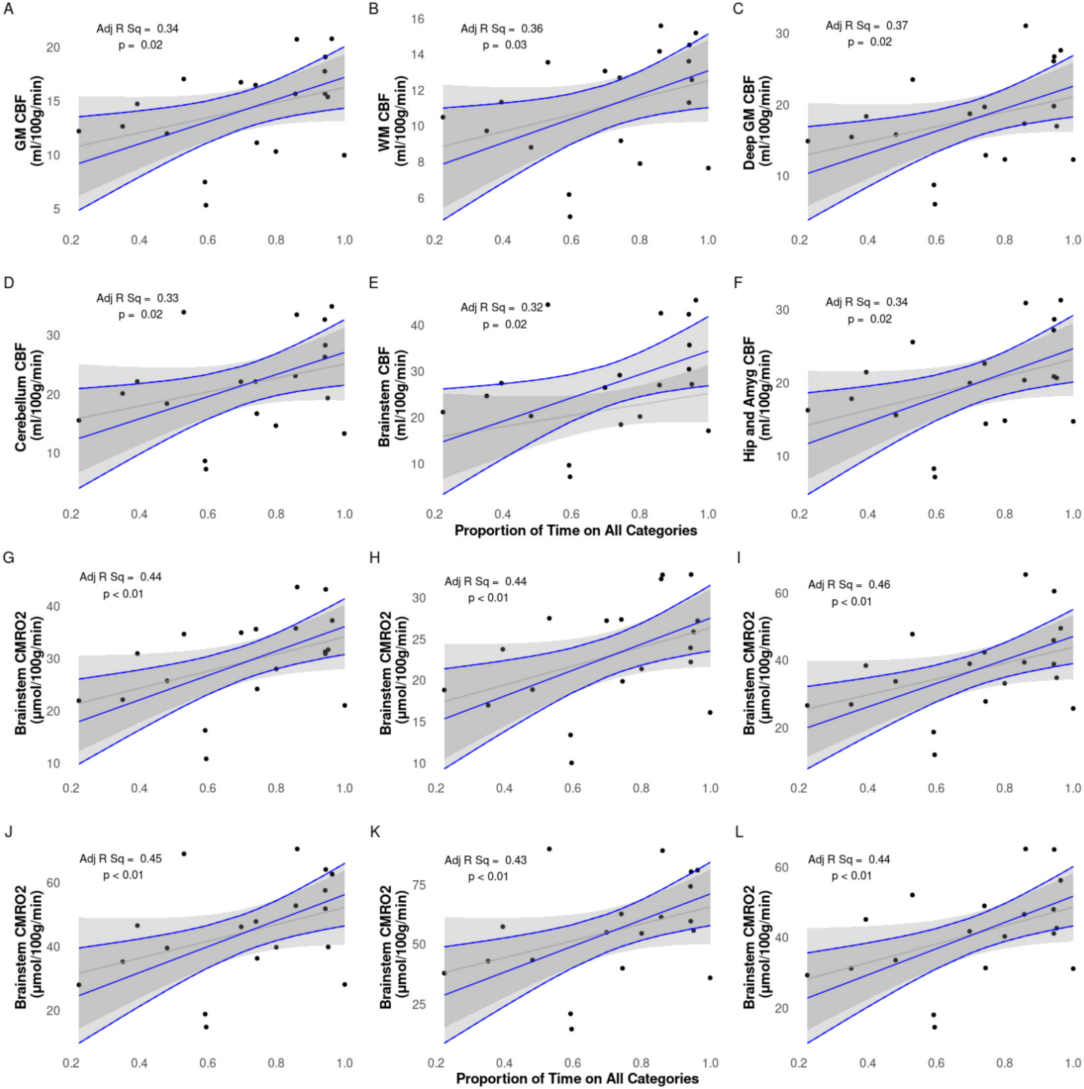
CBF (A–F) and CMRO_2_ (G–L) vs. proportion of time on all categories while in the NICU. Raw data points as filled black circles. Grey line and ribbon represent linear model of raw data points and 95% interval, respectively. Blue line and ribbon represent adjusted multiple linear regression including GA and PMA as confounding factors.

**FIGURE 6:**
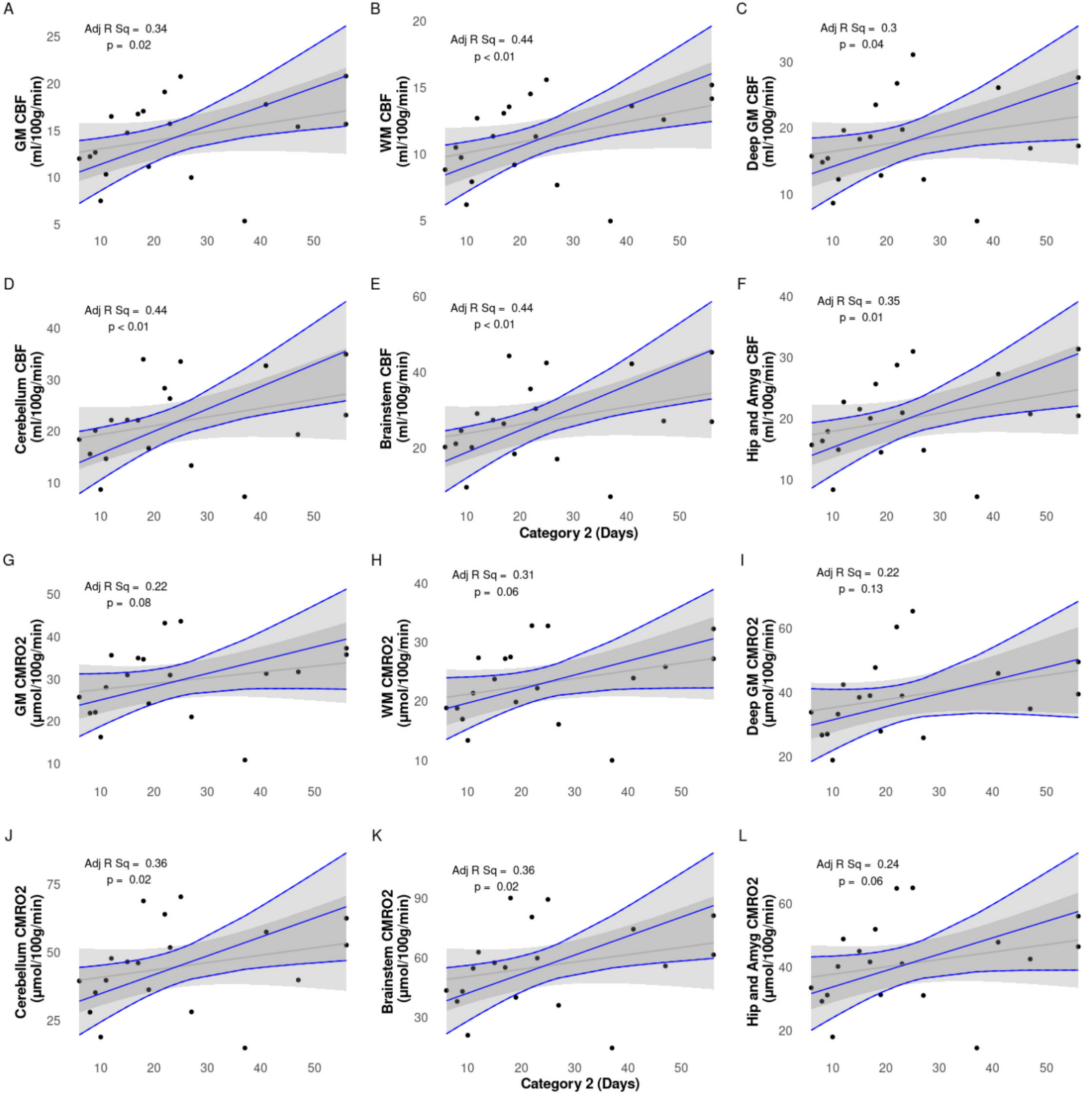
CBF (A–F) and CMRO_2_ (G–L) values against days on noninvasive ventilation (Category 2). Raw data points as filled black circles. Grey line and ribbon represent linear model of raw data points and 95% interval, respectively. Blue line and ribbon represent adjusted multiple linear regression including GA and PMA as confounding factors.

**FIGURE 7:**
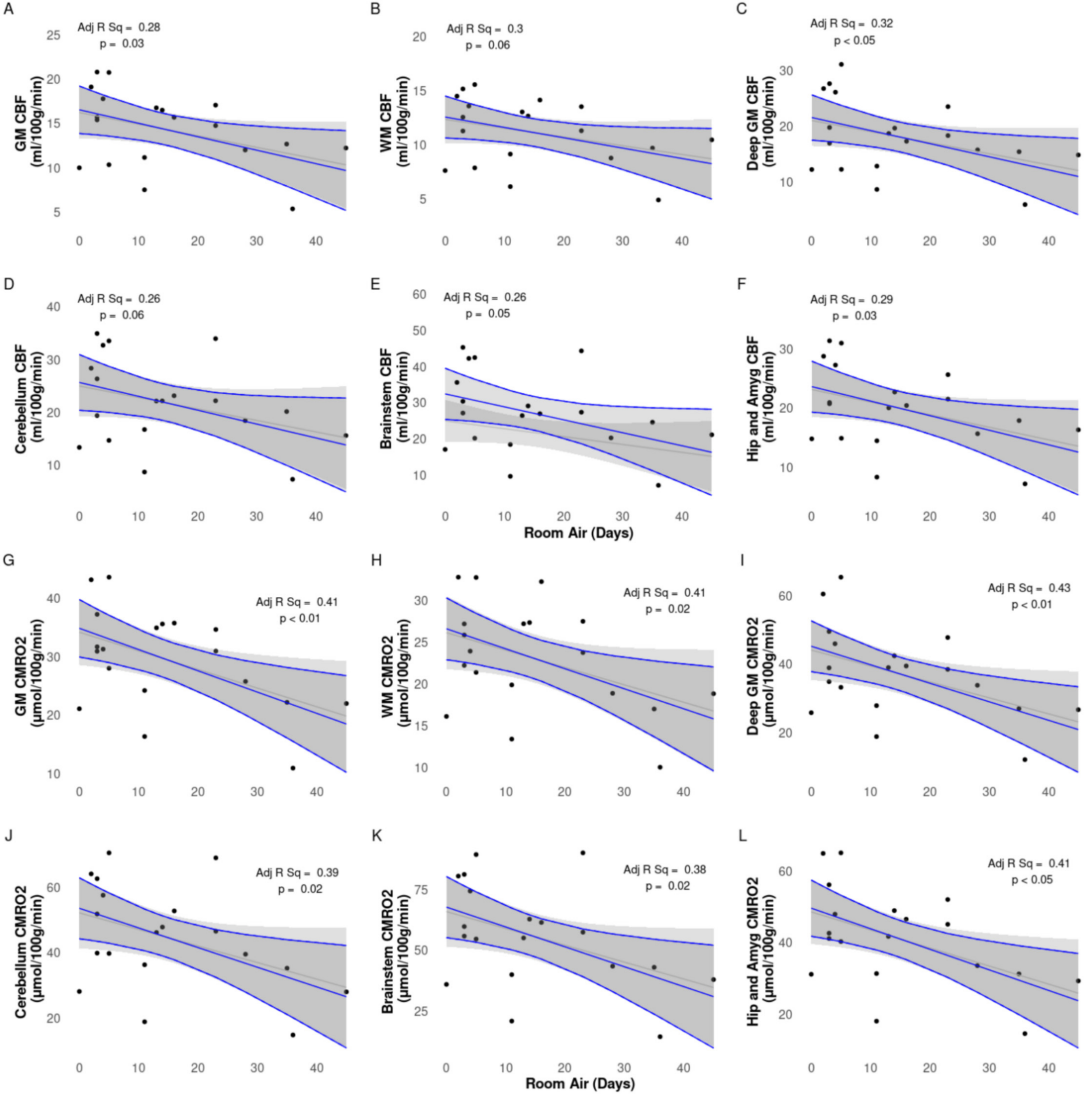
CBF (A–F) and CMRO_2_ (G–L) values vs. days in room air. Raw data points as filled black circles. Grey line and ribbon represent linear model of raw data points and 95% interval, respectively. Blue line and ribbon represent adjusted multiple linear regression including GA and PMA as confounding factors.

Multiple linear regression analysis of semi‐regional CMRO_2_ and regional CBF showed significant positive correlations with proportion of days on respiratory support (Figure 5), days on Category 2 respiratory support (Figure 6), and significant negative correlations with days in room air (Figure 7). Results are summarized in Table 3.

**TABLE 3:** Results from linear regression of physiological parameters with days on various forms of respiratory support.

ROI	Invasive ventilation (Category 1)	Noninvasive ventilation (Category 2)	High‐flow and low‐flow air (Category 3)	Proportion of days on all categories	Room air
	β [95% CI]/Adj R^2^	β [95% CI]/Adj R^2^	β [95% CI]/Adj R^2^	β [95% CI]/Adj R^2^	β [95% CI]/Adj R^2^
CBF					
CGM	0.05 [−0.31 to 0.41]/0.02	**0.2 [0.05 to 0.36]/0.34**	0.13 [−0.14 to 0.4]/0.08	**10.27 [2.17 to 18.37]/0.34**	**−0.15 [−0.29 to −0.02]/0.28**
WM	0.08 [−0.17 to 0.32]/0.13	**0.15 [0.05 to 0.26]/0.44**	0.07 [−0.12 to 0.26]/0.14	**6.7 [0.86 to 12.55]/0.36**	−0.1 [−0.19 to 0]/0.3
DGM	−0.05 [−0.59 to 0.5]/0.06	**0.28 [0.02 to 0.53]/0.3**	0.16 [−0.26 to 0.57]/0.1	**15.82 [3.51 to 28.13]/0.37**	**−0.23 [−0.44 to −0.03]/0.32**
Cereb	0.18 [−0.5 to 0.87]/0.07	**0.43 [0.14 to 0.72]/0.44**	0.21 [−0.31 to 0.73/0.09	**18.81 [2.98 to 34.64]/0.33**	−0.26 [−0.53 to 0.01]/0.26
BS	0.17 [−0.75 to 1.09]/0.05	**0.59 [0.2 to 0.97]/0.44**	0.21 [−0.49 to 0.92]/0.06	**25.22 [3.9 to 46.53]/0.32**	−0.36 [−0.72 to 0]/0.26
H&A	0.05 [−0.53 to 0.63]/0.02	**0.33 [0.08 to 0.59]/0.35**	0.13 [−0.31 to 0.57]/0.04	**16.73 [3.73 to 29.73]/0.34**	**−0.25 [−0.47 to −0.03]/0.29**
CMRO_2_					
CGM	0.04 [−0.68 to 0.77]/0.04	0.31 [−0.04 to 0.66]/0.22	0.25 [−0.29 to 0.79]/0.1	**23.31 [8.19 to 38.44]/0.44**	**−0.36 [−0.62 to −0.11]/0.41**
WM	0.13 [−0.39 to 0.64]/0.14	0.24 [−0.01 to 0.48]/0.31	0.15 [−0.24 to 0.54]/0.16	**15.68 [4.38 to 26.98]/0.44**	**−0.36 [−0.62 to −0.11]/0.41**
DGM	−0.19 [−1.27 to 0.89]/0.09	0.41 [−0.13 to 0.95]/0.22	0.3 [−0.52 to 1.12]/0.12	**34.74 [11.97 to 57.5]/0.46**	**−0.54 [−0.92 to −0.16]/0.43**
Cereb	0.27 [−1.03 to 1.56]/0.1	**0.7 [0.11 to 1.29]/0.36**	0.39 [−0.59 to 1.38]/0.13	**40.71 [12.88 to 68.54]/0.45**	**−0.6 [−1.08 to −0.12]/0.39**
BS	0.2 [−1.55 to 1.96]/0.07	**0.96 [0.17 to 1.75]/0.36**	0.4 [−0.95 to 1.74]/0.09	**54.63 [16.85 to 92.4]/0.43**	**−0.82 [−1.46 to −0.18]/0.38**
H&A	0 [−1.15 to 1.15]/0.04	0.52 [−0.03 to 1.07]/0.24	0.25 [−0.63 to 1.13]/0.06	**37.23 [13.28 to 61.17]/0.44**	**−0.58 [−0.98 to −0.17]/0.41**
CSvO_2_ (%)					
Whole brain	−0.16 [−0.51 to 0.2]/−0.1	0.06 [−0.14 to 0.25]/−0.13	−0.06 [−0.34 to 0.22]/−0.15	−0.68 [−10.72 to 9.36]/−0.16	−0.02 [−0.19 to 0.14]/−0.15
CSaO_2_ (%)					
n/a	−0.05 [−0.18 to 0.08]/0.06	−0.06 [−0.12 to 0]/0.23	0.02 [−0.08 to 0.12]/0.03	0.34 [−3.23 to 3.91]/0.02	−0.01 [−0.07 to 0.05]/0.02
OEF (%)					
Whole brain	0.13 [−0.26 to 0.52]/−0.14	‐0.1 [−0.31 to 0.11]/−0.1	0.07 [−0.23 to 0.38]/−0.15	0.83 [−10.03 to 11.68]/−0.17	0.02 [−0.16 to 0.19]]/−0.17
Hct (%)					
n/a	−0.19 [−0.49 to 0.12]/−0.07	−0.02 [−0.2 to 0.15]/−0.18	−0.05 [−0.3 to 0.19]/−0.17	2.35 [−6.48 to 11.17]/−0.16	−0.09 [−0.23 to 0.05]/−0.05

*Note:* All analyses included GA and PMA as confounding variables.

Abbreviations: BS = brainstem; Cereb = cerebellum; CGM = cortical grey matter; DGM = deep grey matter; H&A = hippocampus and amygdala; WM = white matter.

No significant relationships were found between respiratory support categories and OEF, CSvO_2_, CSaO_2_, and Hct (Table 3). No significant relationship was observed between brainstem volume and days on Category 1 respiratory support.

## 4 Discussion

We presented the initial results of a novel, noninvasive MRI method and analysis pipeline to evaluate CSvO_2_, OEF, and CMRO_2_ in preterm neonates. The values we found agreed well with earlier reported reference values. In addition, our technique allowed us to examine the effects of various forms of respiratory support on CBF and CMRO_2_ in neonates born very preterm. We found that the proportion of days on respiratory support was positively associated with both CBF and CMRO_2_ in all brain regions, a negative association was found for both CBF (some brain regions) and CMRO_2_ (all brain regions) with days in room air, and noninvasive ventilation showed a positive association with CBF in all regions, and a positive association with CMRO_2_ in the brainstem and cerebellum.

### 4.1 Comparison of MRI Methods With Previous Literature

The results from previous neonatal studies are summarized in Table 4. The global CMRO_2_, CBF, OEF, and CSvO_2_ values from this study align well with the literature from MRI, NIRS, and PET studies reported for TEA infants.

**TABLE 4:** Comparison of oxygenation and cerebral hemodynamics across studies.

Study	Method	Number of subjects	PMA (weeks)	CSaO_2_ (%)	CSvO_2_ (%)	OEF (%)	CBF (mL/100 g/min)	CMRO_2_ (μmol/100 g/min)
This study	MRI	19 preterm at term	40.4 ± 1.4	98.3 ± 1.5	63.9 ± 4	34.9 ± 4.3	14.3 ± 4.2	29.5 ± 8.6
Altman et al. (1993)	PET	11 HIE and other conditions	35.1 ± 6.2	N/A	21.6 ± 21.0	16.6 ± 13.2	21.6 ± 21.1	21.4 ± 16.4
De Vis et al. (2014)	MRI	10 preterm at term	39	97 ± 1	52 ± 12	49 ± 12	14 ± 3	30 ± 6
		9 HIE	38	96 ± 3	65 ± 13	32 ± 12	12 ± 4	24 ± 12
Liu et al. (2014)	MRI	12 healthy	37.4 ± 2.6	95.8 ± 2.2	62.6 ± 8.3	N/A	13.4 ± 4.2	38.3 ± 17.7
Elwell et al. (2005)	NIRS	9 ventilatory support	29.2 ± 5.3	N/A	N/A	N/A	N/A	45.9 ± 12.3
Skov et al. (1993)	NIRS	10 asphyxiated (full term)	38.8 ± 1.4	94 ± 7	67.3 ± 9.4	28.4 ± 0.3	26.5 ± 17.9	62.6 ± 35.8
		22 RDS (preterm)	29.8 ± 2.6	96.5 ± 5	53.4 ± 15.4	44.6 ± 2.1	11.9 ± 5.2	44.7 ± 17.9
Yoxall & Weindling (1998)	NIRS	9 ventilatory support	23–37	N/A	62.6 ± 8.3	N/A	13.4 ± 4.2	38.3 ± 17.7
Qi et al. (2018)*	MRI	38 healthy	35.71 (5.36)	95 (2)	63.85 (4.8)	32.35 (6.26)	15.35 (9.13)	38.09 (18.84)
		23 PWML	35.14 (3.29)	95 (2)	68.7 (16.3)	27.53 (18.94)	12.63 (7.83)	29.11 (16.8)

*Note:* This table compares arterial and venous oxygenation, oxygen extraction fraction, cerebral blood flow, and cerebral metabolic rate of oxygen from the present study with findings from previous research.

Abbreviations: BS = brainstem; Cereb = cerebellum; CGM = cortical grey matter; DGM = deep grey matter; H&A = hippocampus and amygdala; WM = white matter.

* Median and interquartile range reported.

One strength of using ASL compared with similar studies that used phase‐contrast to calculate CBF is the ability to look at regional changes in CBF as opposed to a single number for the whole‐brain [19, 20]. This is best demonstrated in the difference we see when looking at correlations with Category 2, where all regions were found to have a positive correlation with CBF, but only the brainstem and cerebellum were found to be positively correlated with CMRO_2_. This discrepancy is discussed more below. However, using ASL in infants also has drawbacks that should be considered, including low signal‐to‐noise ratio, quantification difficulties due to uncertainty in labelling efficiency and bolus arrival time, and the rapid changes that occur in such young populations that make single‐imaging‐protocol difficult [58]. Another method future researchers should consider is quantitative blood oxygenation level‐dependent imaging (qBOLD) [59].

Similarly, a strength of using QSM to study CSvO_2_ rather than previous MRI methods that used the TRUST [19, 20] or T_2_‐TRIR [26], is that QSM produces a whole‐brain map with high spatial resolution. By producing a whole‐brain map, we were able to measure the CSvO_2_ by averaging over all internal veins. This is likely to produce a more robust measurement than acquiring a single slice and averaging within the superior sagittal sinus (SSS) as TRUST and T_2_‐TRIR do. For the current study, our QSM maps were reconstructed to a 0.9 × 0.9 × 0.9 mm^3^ resolution, but future studies would benefit from acquiring and reconstructing up to 0.5 × 0.5 × 0.5 mm^3^. Indeed, greater spatial resolution would likely improve CSvO_2_ measurements as χ values could be better isolated to venous tissue without including nonvenous sources. Finally, higher resolution QSM could also allow for fully regional analysis of CSvO_2_ values, which we did not attempt here. Unfortunately, as our method for calculating QSM requires removing brain tissue along the edge of the brain (an eroded brain mask), we could not measure CSvO_2_ values in the SSS for more direct comparisons. Future work should be directed at acquiring QSM values in the SSS.

Two of the studies that measured CMRO_2_, CBF and CSvO_2_ in sick newborns requiring ventilatory support did not investigate associations between these values and days on various forms of respiratory support. Therefore, we were not able to directly compare these findings.

### 4.2 Respiratory Support

In the present study, more days in room air without any type of respiratory support was associated with lower CMRO_2_ and CBF values. If the assumption of higher CMRO_2_ and CBF are indications of more optimal brain health, then this may suggest that the use of some form of respiratory support may be more beneficial to very preterm infants than weaning to room air. Indeed, CMRO_2_ and CBF were positively related to the proportion of time on respiratory support compared with the total time in the NICU. However, caution must be advised when interpreting these findings. Our study was observational, therefore, we cannot exclude various confounding factors, such as various levels of illness which would have dictated the form of respiratory support the infant received. We may be observing a compensatory effect, wherein infants who were sicker may have over‐compensated for CBF and CMRO_2_ to provide adequate oxygen. This would imply that increased CMRO_2_ and CBF are a reflection of higher illness severity. It is critical for future studies to further explore this relationship because, if on the other hand, being in room air indicates suboptimal cerebral oxygenation and metabolism, it may significantly influence how infants in the NICU are managed.

Furthermore, while all brain regions were found to have a negative correlation between days in room air and CMRO_2_, this was not the case for CBF, where only the cortical grey matter, deep grey matter, and hippocampus & amygdala were found to be negatively correlated with days in room air. Specific brain structures appear to regulate the level of CBF differently and independently of CMRO_2_, suggesting that these brain regions may be more susceptible to or protected from hypoxia. Indeed, evidence for physiological uncoupling of CBF and CMRO_2_ has been reported previously [60, 61, 62]. However, caution should be exercised when drawing strong conclusions from our exploratory analysis.

Invasive ventilation was not found to have to be associated with CMRO_2_ or CBF in any tissue regions. This was unexpected as we hypothesized that infants who required more days on invasive ventilatory support would have lower CMRO_2_ values at TEA. We also did not find a relationship between invasive mechanical ventilation and brain stem volume at TEA, unlike a previous study by Guillot et al. (2020) [7]. Our results are likely limited by the low exposure of this population to invasive mechanical ventilation, as only one infant required invasive support for a prolonged period of time (> 28 days).

Noninvasive ventilation support was associated with increased CMRO_2_ and CBF in preterm neonates at TEA. The observed increase in CMRO_2_ and CBF with noninvasive ventilation *suggests* that prioritizing noninvasive over invasive ventilation may improve brain health outcomes in preterm neonates. However, caution should be exercised as our findings are exploratory in nature. Future studies on respiratory support strategies should incorporate CMRO_2_ and CBF measurements to better understand their relationship with cerebral oxygenation and include a healthy term control cohort to establish comparative baseline values.

The difference between elevated CMRO_2_ and CBF was also observed within specific tissues where CBF increased in all tissues for infants on noninvasive ventilation, but CMRO_2_ only increased in the brainstem and cerebellar tissue. One possibility for this observation could be that compensatory mechanisms are activated in response to respiratory distress or regional brain injuries that hinder the uptake of oxygen. As with our findings in room air, this suggests that specific brain regions respond to respiratory support differently and may be more susceptible to damage. However, further research is first required to reproduce our exploratory findings.

### 4.3 Limitations

There are several limitations that are worth highlighting. Our CSvO_2_ processing pipeline filtered out χ values below 0.15 ppm in order to obtain realistic values. Furthermore, our SWI sequence was acquired at 1 × 1 × 2 mm^2^ but were reconstructed to 1 × 1 × 1 mm^3^ through zero‐padded Fourier interpolation. This interpolation may introduce partial volume effects, particularly in small venous structures in neonates. Future studies should use smaller voxel sizes acquired isotropically, as well as a technique to decompose paramagnetic and diamagnetic values in order to avoid this step [63]. See a recent study of ours for an attempt at this technique [64]. Furthermore, in order to reduce QSM artifacts, the exterior of the brain mask was eroded, making it impossible to measure CSvO_2_ in the SSS. Future studies may find a way to measure QSM in the SSS, which would also allow researchers to determine if CSvO_2_ values are different in the SSS compared with the central cerebral veins. Again, see a recent study of ours that attempted this [64]. CSvO_2_ values were not regional, but were averaged from the central cerebral veins. Future studies should look into ways of creating a fully‐regional voxel‐by‐voxel CSvO_2_ map. One source of inspiration could be Kudo et al. (2015), who created OEF maps from QSM data by using a local threshold method with a volume‐of‐interest (VOI) of 50 × 50 × 50 mm^3^ [65]. However, this would result in a low resolution image. Hct levels were not collected on the day of the scan, but instead were predicted based on past values. Our respiratory support analysis was exploratory with a small sample size. Thus, the positive correlation we found in Category 2 may be spurious or a result of an unaccounted factor. Future studies using large sample randomized controlled trials would provide a clearer understanding of the relationship between respiratory support and cerebral oxygenation. Imaging was performed at term‐equivalent age after the infants had been discharged from the NICU, meaning the scans were performed weeks after the infants were last on respiratory support. Obtaining scans while the infants are still receiving respiratory support could provide a more robust mechanistic connection. Our study did not include a healthy control cohort to compare the expected physiological measures at TEA. This would be important to include, as too much oxygen can be just as harmful as not enough [66]. Finally, due to our sample size, we were unable to explore supplementary variables, such as medications, that may affect respiratory uptake and oxygen metabolism, and patterns of oxygen saturations infants experience during their NICU stay. We see our study as a first step, and that the data shows promise to direct a larger study where these confounds can be addressed.

## Supporting information


**Figure S1:** Predicted Hct value (red) from previously acquired values (black) using a four‐parameter Weibull function from a sample subject. X axis is day of year and y axis is Hct values (fraction).

## Data Availability

The manuscript was written in a ‘reproducible manner’. The entire manuscript, including statistics reported, figures, and tables, can be reproduced at weberlab.github.io/CMRO2_Manuscript/. The data that support the findings of this study are available on request from the corresponding author. The data are not publicly available due to privacy or ethical restrictions.
